# Cancer of Breasts and Ovary

**Published:** 1902-10-25

**Authors:** 


### CANCER OF BREASTS AND OVARY.

The very curious if not absolutely unique specimen exhibited by Mr. Bland-Sutton at the last meeting of the Medical Society illustrated among other things how completely a cancerous infection may keep to its type even though implicating far distant organs, and also raised the interesting question whether a second growth occurring in the opposite breast to that in which the first cancer had arisen should be regarded as a secondary cancer or as an independent growth. In 1893 Mr. Bland-Sutton recognised in his patient's right breast an undoubted cancer. His diagnosis was confirmed by others, and he removed the breast, the pectoral fascia, and the axillary lymph glands with the surrounding fat in January 1894. Three years later a hard nodule appeared in the scar, and was promptly removed. In another year another nodule appeared in the scar. This was allowed to remain for several months, until it ulcerated, when the patient became alarmed and went again to Mr. Bland-Sutton, who removed it without delay. Five years and a half from the date of the primary operation the patient had developed an enormous mass of cancer in the left breast, with enlargement of the axillary lymph glands. This breast and glands were removed without delay. Recovery was prompt and satisfactory and all went on well until January of this year, when a tumour as large as a cricket ball was discovered in the abdomen. This grew very rapidly, fluid formed in the peritoneum, and in March this tumour was removed. It proved to be a large ovoid tumour of the right ovary. It was extremely dense, and might be compared in size to a large cocoa nut. On section it was found to be solid throughout, and on microscopical section displayed the structure of mammary cancer in its most typical form. During the operation the left ovary was removed also in order that the patient might have any advantage which might be derived from the effect of a double ovariotomy upon the cancer process. Commenting upon this case Mr. Bland-Sutton remarked upon the rarity with which cancer attacked both breasts either concurrently or with an interval perhaps of years. On several occasions he had watched patients in the Cancer Asylum of the Middlesex Hospital in whom he had suspected secondary deposits in the breast, but when it became possible to verify the observations the suspected nodules were found not to be cancerous, and he had come to the conclusion that when a woman had a breast removed for cancer she was five times more likely to have secondary deposits of cancer in one or both ovaries than in the remaining breast.

